# Macro-Size Regenerated Cellulose Fibre Embedded with Graphene Oxide with Antibacterial Properties

**DOI:** 10.3390/polym15010230

**Published:** 2023-01-01

**Authors:** Nyak Syazwani Nyak Mazlan, Kushairi Mohd Salleh, Mohamad Khalid Khairunnisa-Atiqah, Abdul Hair Ainul Hafiza, Marhaini Mostapha, Amanda V. Ellis, Sarani Zakaria

**Affiliations:** 1 Bioresource and Biorefinery Laboratory, Faculty of Science and Technology, Universiti Kebangsaan Malaysia, Bangi 43600, Malaysia; 2 Bioresource Technology Division, School of Industrial Technology, Universiti Sains Malaysia, Penang 11800, Malaysia; 3 Renewable Biomass Transformation Cluster, School of Industrial Technology, Universiti Sains Malaysia, Penang 11800, Malaysia; 4 Centre of Foundation Studies, Universiti Teknologi MARA, Cawangan Selangor, Kampus Dengkil, Dengkil 43800, Malaysia; 5 Centre of Health Economic Research, Institute Health System Research, National Institute of Health Malaysia, Shah Alam 40171, Malaysia; 6 Department of Chemical Engineering, The University of Melbourne, Grattan Street, Parkville, VIC 3010, Australia

**Keywords:** Bioresource based products, NaOH/urea, regenerated cellulose, solution mixing method, green product, production

## Abstract

Macro-size regenerated cellulose fibres (RCFs) with embedded graphene oxide (GO) were fabricated by dissolving cellulose in a pre-cooled sodium hydroxide (NaOH)/urea solution and regenerated in sulphuric acid (H_2_SO_4_) coagulant. Initially, GO was found to disperse well in the cellulose solution due to intercalation with the cellulose; however, this cellulose–GO intercalation was disturbed during the regeneration process, causing agglomeration of GO in the RCF mixture. Agglomerated GO was confirmed at a higher GO content under a Dino-Lite microscope. The crystallinity index (CrI) and thermal properties of the RCFs increased with increasing GO loadings, up to 2 wt.%, and reduced thereafter. Cellulose–GO intercalation was observed at lower GO concentrations, which enhanced the crystallinity and thermal properties of the RCF–GO composite. It was shown that the GO exhibited antibacterial properties in the RCF–GO composite, with the highest bacterial inhibition against E. coli and S. aureus.

## 1. Introduction

Natural polymers have been widely studied for their outstanding properties, such as biodegradability [1], recyclability, non-cytotoxicity, and much more. Cellulose is the most abundant natural polymer on Earth; therefore, it can be used as a potential replacement for petroleum-based polymers. The majority of industries that are based on agricultural production or forest products generate waste in the form of biomass as a by-product; however, this waste is not currently being utilised to its full potential. It has been demonstrated that cellulose biomasses can be utilised in a variety of specialised products, including membranes [2,3,4], hydrogels [5,6,7], aerogels, and fibres [8,9]. The chemical, physical, and mechanical properties of cellulose are all amenable, simple chemical treatments. Cellulose II forms: (1) when cellulose I is treated with sodium hydroxide, (2) when cellulose I is precipitating from a solution of alkali/salt, or (3) by removing the added functional groups from cellulose derivatives. These treatments change the lattice structure of cellulose I to cellulose II [2,10]. Products made from regenerated cellulose can be manufactured into a wide variety of forms, and their potential uses are virtually endless. These products include food packaging materials [11,12,13], composites for the automobile industry [14], construction, and medical devices [15,16,17].

The unique properties of cellulose make it a preferred material in biomedical applications because it is biocompatible, non-toxic, and produces no harmful by-products when it breaks down inside the human body. The utilisation of cellulose-based materials and products in biomedical applications, such as drug delivery systems, bio-scaffolds, and wound dressing, is rapidly increasing. For a wound to have a healthy wound-healing process, the treated region must be moist enough for cell proliferation [18]. It is well known that cellulose is hydrophilic and can retain high moisture content. The presence of moisture, on the other hand, makes it easier for microorganisms, such as bacteria and fungi, to proliferate and spread, which can result in wound infection. Cellulose-based materials are highly susceptible to microorganisms, especially bacteria and fungi, which prematurely degrade cellulose. In order to use cellulose for biomedical applications or anything comparable to them, antimicrobial properties need to be incorporated into the material.

Previous literature has shown that graphene oxide (GO) can impart antimicrobial activity and prolong the functionality of a wound dressing [19]. GO is a derivative of graphene, produced from the chemical exfoliation of graphite to form a thin two−dimensional (2D) sheet that contains oxidative functional groups, such as hydroxyl (–OH), epoxy (COC), carbonyl (–C=O), and carboxylic acid (–COOH) [20]. The presence of hydrophilic oxygen-containing groups and the hydrophobic basal plane of GO can react covalently or non-covalently with different compounds [21,22]. These interactions can help with the distribution and bonding of GO particles throughout a polymeric matrix, such as cellulose.

GO has been utilised for an anti-adhesive effect on bacterial colonisation and kills bacteria upon contact to prevent the microorganism colonisation that would otherwise occur [23]. Due to the fact that GO has a 2D structure, its general antipathogenic mechanism in systems is to cause cell membrane damage, disruption of phospholipids, charge transfer, the production of reactive oxygen species (ROS), and oxidative stress [24]. According to the findings of various studies published in the scientific literature, GO can eradicate bacteria by severing the cell membrane with the GO-oxygenated edge planes. GO’s thin sheets have the potential to function as a nano-knife or nano-blade, resulting in the death of microorganisms due to the leaking of intracellular constituents [23].

The incorporation of GO into celluloses may also influence composite material’s mechanical and physical properties. In most cases, GO is incorporated into a polymer matrix as a reinforcement material to improve the polymer’s mechanical properties [25].

In the current study, we investigate whether or not GO is immiscible or compatible with an alkaline-dissolved cellulose solution, as well as the extent to which it is possible to incorporate GO into a cellulose matrix. To evaluate the compatibility of cellulose–GO and further investigate its potential as an antimicrobial agent, a regenerated cellulose fibre (RCF) was manufactured. GO was reinforced in the cellulose matrix and formed a macro-size RCF via a wet-spinning process. The dispersion and compatibility of GO in a cellulose solution and the RCF were investigated. The physical and mechanical properties of the final RCF–GO composite were also evaluated. Antibacterial testing was conducted on the RCF formed from NaOH/urea system. This invention is fascinating in that it highlights the potential use of RCFs as a biocompatible wound dressing, has low toxicity, is non-allergenic, and contains antibacterial properties within the material.

## 2. Materials and Methods

### 2.1. Materials

Cotton linter pulp was provided by Hubei Chemical Fibre Co. Ltd. (Xiangfan, China). Its viscosity-average molecular weight (Mƞ) was 9.0 × 10^4^ g mol^−1^. GO with a concentration of 6.9 mg mL^−1^ was prepared and provided by Melbourne University (Melbourne, Australia) using the Hummer method. Sodium hydroxide (NaOH), urea, and sulphuric acid (H_2_SO_4_) were purchased from R & M (Southhampton, UK). All chemical reagents were analytical grade and used without further purification.

### 2.2. Preparation of Cellulose/GO Fibre

Precooled alkaline solution was prepared with a weight ratio of 7:12:81 (NaOH/urea/GO). Cotton linter pulp (4 g) was dissolved in an aqueous NaOH/urea solution via rapid dissolution. The resulting solution was then vigorously stirred for 5 min to form a homogeneous cellulose solution. The cellulose solution was centrifuged for degassing and separating the dissolved and undissolved cellulose. The dissolved cellulose solution was removed and placed in an ice bath to which different percentages of GO (0–3 wt.%) were added, with stirring, to form a homogeneous solution. 

**Figure 1:**
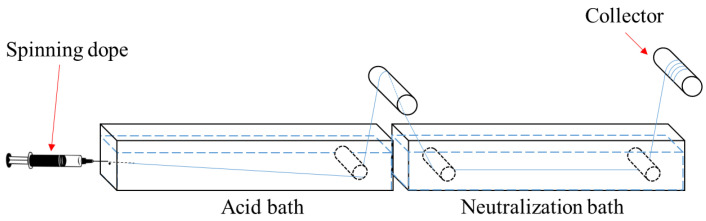
Illustration of in-house wet-spinning setup.

Cellulose–GO fibre (RCF) was fabricated using an in-house wet-spinning setup with 7 wt.% H_2_SO_4_ as a coagulant, as illustrated in Figure 1. The wet-spinning setup was divided into two sections, coagulation (regeneration) and washing (neutralisation). The cellulose solution (spinning dope) was extruded through a blunt needle (size, 20D) directly into the coagulant (acid, H_2_SO_4_) bath at a rate of 50 mL/h. The formed RCF was immersed in the coagulant for 5 s before being transferred into a distilled water bath (neutralisation bath) and kept there for 15 min. The RCF was rinsed three times in distilled water to remove the excess NaOH/urea solution and the coagulant before being dried under an infrared drier at 125 °C to form macro-thickness fibres for further characterisation. Samples were labelled as RCF−0 (0 wt.% GO), RCF−GO1 (1 wt.% GO), RCF−GO2 (2 wt.% GO), and RCF−GO3 (3 wt.% GO).

### 2.3. Raman Spectroscopy

Several drops of the GO dispersion in water were placed on a glass slide and allowed to air dry. Raman scattering spectra for the GO were obtained from 1000 to 2000 cm^−1^ using Raman micro-spectroscopy (uRaman-Ci, Technospex, Singapore), with a laser excitation of 532 nm at 0.1 mW laser power for a 10 s exposure time.

### 2.4. Transmission Electron Microscopy (TEM)

GO in distilled water was placed on a glass slide and air dried to obtain dried GO particles. The morphological structure and the shape of GO were observed using TEM (TEM-Philips, CM-12, Amsterdam, Netherland) at 10,000 magnifications operated at a 100 kV accelerating voltage.

### 2.5. Attenuated Total Reflection-Fourier Transform Infrared (ATR-FTIR) Spectroscopy

ATR-FTIR spectra were measured using a Bruker ATR-FTIR spectrophotometer (Billerica, MA, USA) and analysed from 4000 to 800 cm^−1^ with 32 scans at a resolution set to 4 cm^−1^. RCFs were cut to form powder-like particles. Approximately 0.1 g of the powder was used for ATR-FTIR characterisation.

### 2.6. X-Ray Diffractometry (XRD) Analysis

The X-ray diffraction pattern for all samples was obtained using an XRD (Bruker/ D8 Advance, Billerica, MA, USA) with CuKα (λ = 0.15458 nm). The samples were scanned from 5° to 35° 2θ. The crystallinity index (*CrI*) of the samples was calculated using Equation (1):

(1) $$CrI=\frac{A_{Crystal}}{A_{crystal}+A_{amorphous}}\times 100\%$$

where *A_crystal_* is the area under the crystalline diffraction curve, and *A_amorphous_* is the amorphous area under the diffraction curve

### 2.7. Field-Emission Scanning Electron Microscopy (FESEM)

The image of the cross-section and surface of the RCFs were obtained using a field-emission scanning electron microscope (FESEM Zeiss/Supra 55VP, Oberkochen, Germany). The fibre was freeze-dried and sputter-coated with gold before the FESEM examination at an accelerating voltage of 3.0 kV.

### 2.8. Dino-Lite Microscopy

The images of the RCFs were captured using a Dino-Lite microscope (New Taipei City, Taiwan) at 440× magnification. Approximately 2 cm of RCF was cut and placed on a light background for observation.

### 2.9. Thermogravimetric Analysis 

The thermal analysis of the samples was characterised using a thermal gravimetric analyser (TGA) (Mettler Toledo/ TGA SDTA 851e, Columbus, OH, USA). RCFs were cut into powder-like particles and approximately 8.455 ± 0.355 mg was used for TGA. The conditions for this analysis included a heating rate of 10 °C min^−1^ over the temperature range from 25 to 600 °C in a nitrogen gas atmosphere. 

### 2.10. Mechanical Testing of the RCFs

The mechanical strength of the RCFs was evaluated using a universal testing machine (GOTECH/AI-3000, Taichung, Taiwan) at a rate of 10 mm min^−1^. RCFs were cut into 6 cm lengths (gauge length: 4 cm; grip length: 1 cm). Each grip of the fibre was sandwiched between 2 pieces of 1 × 1 cm of 170 gsm paper to prevent the fibre from slipping during testing. Twelve replicates were made for each sample, and average readings are presented.

### 2.11. Antibacterial Analysis

Antibacterial properties of RCFs with different GO concentrations were conducted using the agar plate diffusion method (GB/T 20944.1-2017), as reported by Fu et al. [26,27]. All samples were sterilised under ultra-violet light for 30 min before use. The bacteria used for this analysis are a clinical strain. Both Gram-negative (*Escherichia coli*) and Gram-positive (*Staphylococcus aureus*) bacteria were diluted in a suspension of 1 × 10^6^ CFU/mL and spread on a sterile agar plate using an inoculating loop. The RCF was placed transverse to the agar surface and incubated at 37 °C for 24 h. The inhibition zones were calculated based on Equation (2):

(2) $$H=\frac{D-d}{d}$$

where *H* is the inhibition zone, *D* is the total diameter of the RCF and the inhibition zone (mm), and *d* is the diameter of the RCF (mm).

## 3. Results

### 3.1. Characterisation of GO

**Figure 2:**
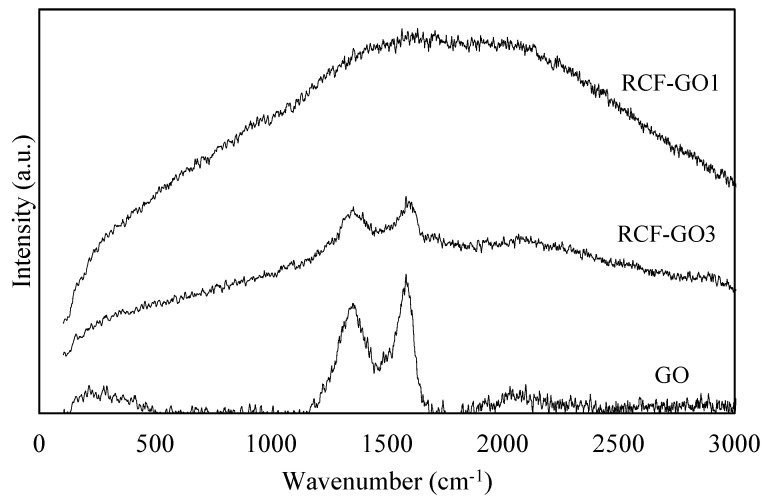
Raman spectrum of graphene oxide (GO), RCF−GO1, and RCF−GO3.

Figure 2 shows that the Raman spectrum of GO indicates two major bands: the D-band at 1351 cm^−1^ and the G-band at 1586 cm^−1^. The D-band is associated with the in-plane bonding–stretching motion of the carbon sp^2^ atom pairs [21,22,28]. In addition, it demonstrates the disordered and randomised structure of the GO, which can be traced back to the graphene sheets. The G-band is associated with the vibration of the sp^2^-bonded carbon atom in the GO [28,29]. When determining how disorderly the GO sheet’s structure is, the intensity ratio of I_D_/I_G_ is an extremely important factor to consider. The intensity ratio I_D_/I_G_ in this study was 0.80. A lower ratio of I_D_/I_G_ (<1) indicates that the carbon is partially crystalline with a disorder in the structure of the carbon atoms [30]. This is attributed to the structural imperfection of the GO sheet with many oxygen-containing groups, which also means the arrangement of the carbon is flawed. Therefore, with an I_D_/I_G_ ratio of 0.80, the GO is expected to have a good amount of oxygen-based functional groups with minimal defects presented, as reported by How et al. [31].

The fact that RCF−GO1 does not exhibit any pronounced peaks suggests that the GO is encapsulated within the cellulose matrix. Due to the low concentration, Raman spectroscopy was not able to detect it, indicating that there was less GO present on the surface of the RCFs. However, RCF−GO3 shows visible bands at 1353 and 1600 cm^−1^ for the D−band and G−band, respectively. The D−band and G−band peaks in RCF−GO3 are less intense than in pure GO because only 3 wt.% of the GO is presented and embedded inside the cellulose matrix. Nonetheless, these peaks verified that GO is also present on the surface of the RCFs’ composite. The I_D_/I_G_ ratio for RCF−GO3 is 0.98, which is lower than that of pure GO. The I_D_/I_G_ ratio is closely related to the crystallinity of the samples. As the crystallinity increases, the I_D_/I_G_ ratio decreases. Given that the GO in RCF−GO3 had intercalated with the cellulose, this result makes sense. As a result, the composite’s crystallinity decreases.

**Figure 3:**
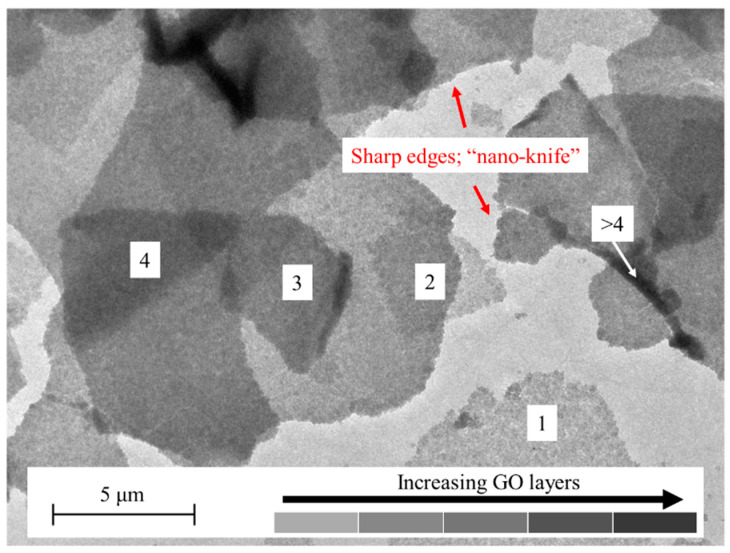
TEM image of the single and multiple layers of “nano-knife” GO in distilled water. Numbers in the figure represent layers of GO.

TEM was used to observe the morphology of the GO at × 10,000 magnification (Figure 3). Different “grey-ish” shades were seen, signifying GO layers, and the GO appeared transparent and sheet-like. In addition, the transparency of the GO showed that the exfoliated GO exists in single and multiple layers. The shade gradients of the GO in Figure 3 were due to different GO sheets stacking on one another. Therefore, higher layers of the GO are presented in a darker shade and vice versa. In addition, the sharp edges of the GO support its potential to act as an antibacterial agent. The sharp edges can impart the “nano-knife” or “nano−blade” effect, disrupting bacteria by cutting the cell membrane upon contact, thus killing the bacteria [19,23].

### 3.2. FTIR Analysis

**Figure 4:**
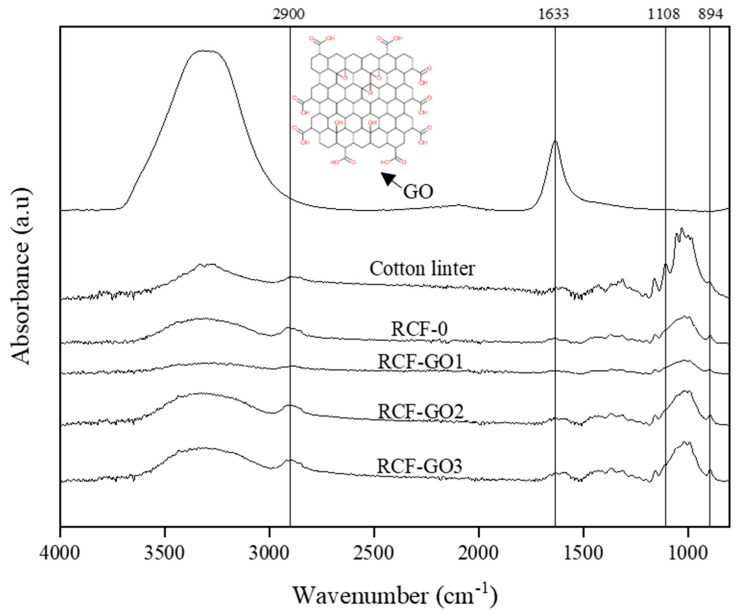
FTIR spectra of GO, cotton linter, RCF−0, and RCF−GO3.

Figure 4 shows the FTIR spectrum of pure GO, cotton linter pulp, RCF−0, RCF−GO1, RCF−GO2, and RCF−GO3. The stretching of the hydrogen-bonded hydroxyl group can be observed in all spectra between 3700 and 3000 cm^−1^. The –OH functional group became broadened for RCF−GO1 upon the addition of GO compared to RCF−0, demonstrating the formation of hydrogen bonding between the cellulose and GO. However, the higher intensity at 3000–3700 cm^−1^ for RCF−GO3 is due to unreacted GO present in the composite. Once the cellulose and GO fully react, the excess GO will still be dispersed in the RCF composite without any chemical interaction. Therefore, the excess GO in the composite may contribute to a higher peak at 3000–3700 cm^−1^ for RCF−GO3. Peak 2900 cm^−1^ represents the amorphous cellulosic samples [32,33]. The peak wavenumbers for CL, RCF−0, RCF−GO1, and RCF−GO3 are 2900, 2916, 2902, and 2916 cm^−1^_,_ respectively. All RCFs shifted to a higher band, indicating an extant amorphous crystalline region [32,33]. 

The sharp peak of the GO at 1633 cm^−1^ corresponds to the double carbon bond (C=C) of the aromatic ring of the GO’s skeleton structure [34]. Furthermore, cotton linter has a small band around 1600 cm^−1^ that represents carboxylic acid stretching in the cellulose. The small band is also present in RCF−0, RCF−GO1, RCF−GO2, and RCF−GO3 with peaks of 1611, 1631, 1627, 1629, and 1629 cm^−1^, respectively. The band slightly shifted to a lower wavenumber, indicating more C = O vibrations detected from the RCF [27]. However, among the RCF composite, RCF−GO1 has the lowest absorption value, followed by RCF−GO2 and RCF−GO3. A higher absorption band contributed to a higher GO concentration in the composite. RCF−0 has a lower peak intensity than CL due to the formation of structured networking in the regenerated cellulose [35].

The asymmetric ring stretching of C–O is seen at 1108 cm^−1^ in the cotton linter pulp, while shoulders are present at the wavenumber 1108 cm^−1^ on RCF−0 to RCF−GO3. However, these shoulders’ intensity reduces compared to those of pure cotton linter indicating the C–O reduces after the cellulose dissolution and regeneration process. This finding indicates that the cotton linter pulp has cellulose I structure, and RCF−0 to RCF−GO3 has a cellulose II structure [35]. Collective peaks at 1027, 1053, and 985 cm^−1^ represent the C–O–C stretching vibration of the pyranose ring skeleton of cellulose. Therefore, these peaks do not exist in GO. Instead, CL has a higher intensity, indicating cellulose I structure rather than regenerated cellulose [36,37]. 

Next, the stretching band at 894 cm^−1^ corresponds to C–O–C at the β-glycosidic linkages in the cellulose. This band also represents the amorphous absorption band. Therefore, the band is more pronounced in the RCF compared to the pure cotton linter [38]. However, the RCF−0 and RCF−GO3 possess larger absorption intensities at 894 cm^−1^, demonstrating a decreased degree of crystallinity in the RCFs, due to the dissolution and regeneration process [33,34]. Based on the FTIR analysis, the physical interaction between the RCF and GO is via intermolecular hydrogen bonding between the cellulose chains of the RCF and the GO. 

### 3.3. Morphology of RCFs

**Figure 5:**
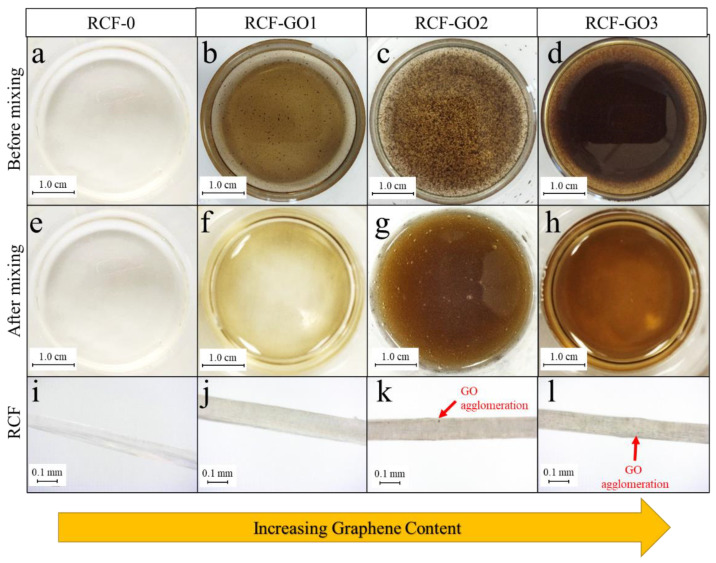
Optical images of (**a**–**c**) GO in cellulose solution prior to regeneration (coagulation); (**e**–**h**) GO in cellulose solution after mixing for 30 min prior to regeneration, and (**i**–**l**) RCF images captured using a Dino-Lite microscope at 470 × magnification.

Figure 5a–d show optical images of the dissolved cellulose with different loadings of GO spinning dope. As the GO loading increases, the cellulose solution changes from yellowish to dark brown. However, the GO became agglomerated with increased loading, as displayed in Figure 5b–d. This phenomenon occurs because GO does not disperse well in highly alkaline media due to the deprotonation of any carboxylic acid groups. Interestingly, however, once GO was stirred in the regenerated cellulose solution for a good 30 min, no agglomeration of the GO was observed, as shown in Figure 5e–h. The darkness on the outer edge was due to the reflection of light. The solution was placed in beakers where the bottom edge is curved. This reflected the light and made the solution seem darker on the edges. An improved GO dispersion in the regenerated cellulose solution is attributed to intercalation between the GO and cellulose, which contributes to good dispersion of the GO in the cellulose solution. This may also be mediated by hydrogen bonding. 

The phenomenon is also confirmed in the fibres (Figure 5i–l). At higher concentrations of GO, a uniform dispersion is observed for RCF−GO2 and RCF−GO3 (Figure 5k,l, respectively). This is a result of the regeneration (coagulation) process in which recrystallisation of the cellulose occurs and GO is intercalated into the RCF’s composite structure. This may happen simultaneously as the regeneration of cellulose occurs on the surface of the fibre and moves inwards to the centre of the fibre. Regardless of the concentration of GO added to the cellulose solution, the RCF’s thickness remained in the macro-range with diameters from 0.83 mm to 1.63 mm.

**Figure 6:**
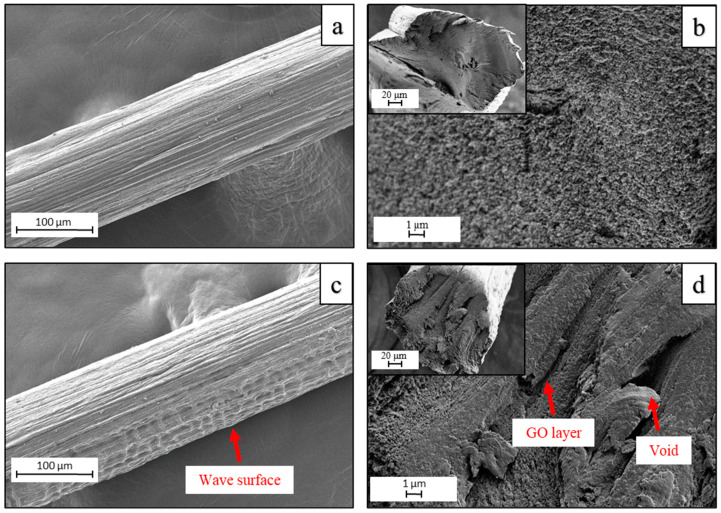
FESEM images of the surface of (**a**) RCF−0 and (**c**) RCF−GO2. Cross-section (inset) and enlargement of (**b**) RCF−0 and (**d**) RCF−GO2.

The surface and cross-sections of RCF−0 and RCF−GO2 were analysed using FESEM (Figure 6, labelled as (a,b) and (c,d), respectively). Both RCFs have longitudinal lines resulting from scratches from the spinneret. The surface of RCF−0 (Figure 6a) is uniform, while RCF−GO2 (Figure 6c) shows a wave-like structure on its surface. The wave-like structure might be caused by the disturbance of the cellulose–GO intercalation during the regeneration process in an acid coagulant. The cross-section of RCF−0 (Figure 6b) appears to have a uniform structure due to cellulose self-aggregation during the regeneration process in the acid coagulant, in the absence of any GO to disturb the cellulose recrystallisation process [39]. Conversely, the cross-section of RCF−GO2 (Figure 6d) shows several micro-voids and layered structuring. The rough cross-section surface of RCF−GO2 (Figure 6d) indicates that the cellulose and GO were not fully unified and exhibited a low homogeneity. This may arise from rapid diffusion during the regeneration process, which interferes with the pre-existing intercalation between the GO and the cellulose.

### 3.4. XRD Analysis

**Figure 7:**
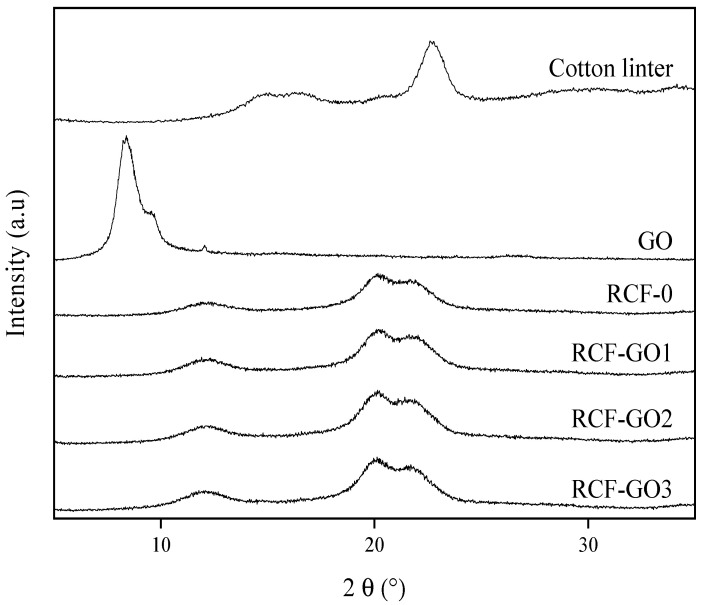
X-ray diffraction diffractograms of cotton linter pulp, RCF−0, RCF−GO1, RCF−GO2, and RCF−GO3.

Figure 7 shows the XRD diffraction spectra of the cotton linter pulp, RFC−0, RCF−GO1, RCF−GO2, and RCF−GO3. Cotton linter displays cellulose I structure with a sharp peak at 22.2° and broad peaks between 14.7° and 16.3°. Upon the cellulose dissolution and regeneration process, the lattice structure of cellulose changes. All RCFs show diffraction peaks at 2θ = 12.1°, 20.2°, and 21.7°, corresponding to (1 ī 0), (110), and (200), respectively, which represents the lattice structure of cellulose II [39,40]. These peaks are not observed in the cotton linter pulp indicating that cellulose I has been converted to cellulose II [39]. Interestingly, no peak is observed from GO (displayed as 2θ = 8.3°) in any of the RCF–GO samples, regardless of GO being observed in the optical images (see Figure 5). GO only agglomerates in some parts of the RCF but not throughout the RCF. For this reason, no GO peaks are expressed on the XRD diffractogram. This suggests that GO has been fully dispersed (intercalated) into the cellulose matrix, especially for the high GO loadings. 

The *CrI* of the RCF samples was calculated based on Eq. 1 and presented in Table 1. Based on the results, increasing the GO content from 1 wt.% to 2 wt.% in the RCFs increases the *CrI* values, but the value decreased when 3 wt.% GO was used. 

### 3.5. Thermal Analysis

**Figure 8:**
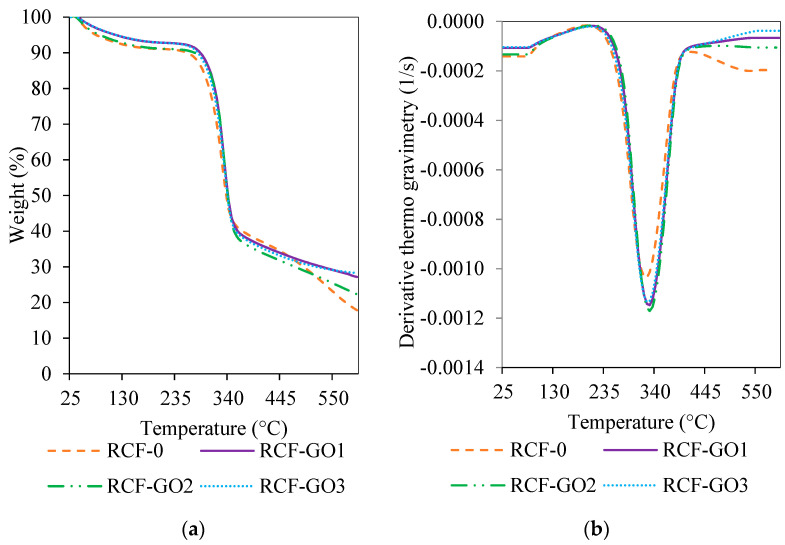
TGA curves of (**a**) RCF–GO with different GO concentrations and (**b**) differential thermogravimetry (DTG) curve of RCF–GO with different GO concentrations.

The thermal properties of the RCFs were characterised using TGA, as displayed in Figure 8. The TGA analysis was conducted to identify the effect of GO loading on the thermal stability of the RCF. Figure 8a shows that there are dehydration and degradation steps associated with the RCF samples. The dehydration step is observed at 100 °C, where most of the water present in the RCF is evaporated. Two types of water are present in most materials: free water and bound water. Free water can be removed at any temperature, and it can also be reabsorbed into a material. However, bound water can only be removed at a temperature higher than 100 °C and cannot be reabsorbed into the material. Next, a degradation step occurs between 275 °C and 345 °C, where the decomposition of the RCF occurs. The detail of the thermal degradation characteristics of RCF (T_o_, T_max_ and T_f_) are listed in Table 2. The onset (T_o_) degradation of RCF–GO increases to a higher temperature up to 30 °C higher than that of RCF–0. This means that the decomposition of the RCF–GO composite is delayed resulting in RCF–GO with higher thermal stability than RCF−0. The degradation temperature increases with the GO content may be contributed by homogenous dispersion of GO throughout the RCF–GO. This improvement is attributed to the restraint of the mobility of cellulose segments at the interface between cellulose and GO. The DTG curve (Figure 8b) for RCF–GO shows a shift to the right compared to RCF−0. All T_o_, T_max_, and T_f_ of the RCF–GO composites are higher than the RCF without GO. Therefore, it can be inferred that the addition of GO has increased the thermal stability of the RCF. This phenomenon may be due to the epoxy, hydroxyl, and carbonyl groups that are presented on the surface of GO interacting with the hydroxyl-rich cellulose, forming new hydrogen bonds [34]. In addition, GO layering in the RCF may lead to an indirect pathway for volatile degradation products, thus delaying the degradation of the whole composite [34] RCF−GO3 showed slight differences. The decomposition temperatures were reduced compared to those of RCF−GO1 and RCF−GO2 (Table 2). This may simply be due to the increased thermal conductivity of the GO at higher loadings.

Figure 8a and Table 2 show that the residue formed increased when GO was present in the RCF. The residue corresponds to the char yield in the RCF–GO, in which GO is a carbon-based material, and its combustion generates a higher char residue.

### 3.6. Mechanical Properties of RCF

**Figure 9:**
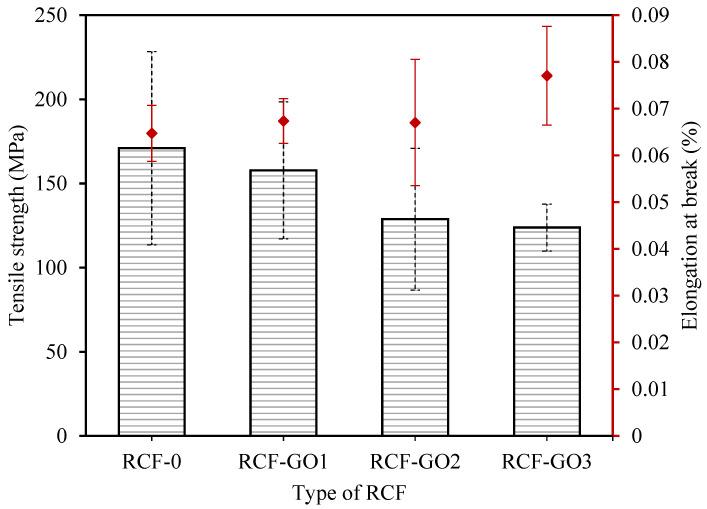
Tensile strength and elongation at break for RCF−0, RCF−GO1, RCF−GO2, and RCF−GO3.

Figure 9 represents the tensile strength and elongation at break for RCFs with and without GO. RCF−0 exhibited the highest tensile strength of 170.96 MPa. Typically, the addition of GO in cellulose fibres should increase the mechanical strength of the RCF [39]. However, in this work, the addition of GO gradually decreased the tensile strength of the RCF. It is noted that the tensile strengths were 157.80 MPa, 128.78 MPa, and 123.82 MPa for RCF−GO1, RCF−GO2, and RCF−GO3, respectively. These values correspond to a decrease of −7.7% to −27.6% relative to RCF−0. 

A higher GO loading appears to weaken the mechanical strength of the RCF. The brittleness of GO ascribes to the occurrence. As observed in images captured using a Dino−Lite microscope in Figure 5k,l, RCFs with higher GO concentrations have visible GO agglomeration in the fibres. It initiates the formation of voids in the fibre structure. GO agglomerates may cause concentrated stress in the fibre resulting in lower mechanical strength of the RCF with GO. Excessive GO also triggers coagulation, causing GO to not fully interact with cellulose. Wang reported that when the amount of GO in cellulose films is greater than 1 wt.%, the mechanical properties of the cellulose–GO composite reduce [39,41]. Similarly, Gan and co-workers reported that increasing the GO loadings to a certain level resulted in a reduction in the mechanical strength of the composite [39]. This illustrates that the reinforcement effect of GO has a close relationship with the dispersion of GO in the polymer and the disturbance of intercalation between cellulose and GO during the regeneration process. The elongation at break of RCF increases as the GO content increases, as seen in Figure 9. This finding may be attributed to higher hydrogen bonding formed between the GO and cellulose, as observed in the FTIR analysis; however, the increment was only approximately 0.02%. Therefore, the addition of GO does not affect the elasticity of RCF.

### 3.7. Antibacterial Activity of RCFs with and without GO

**Figure 10:**
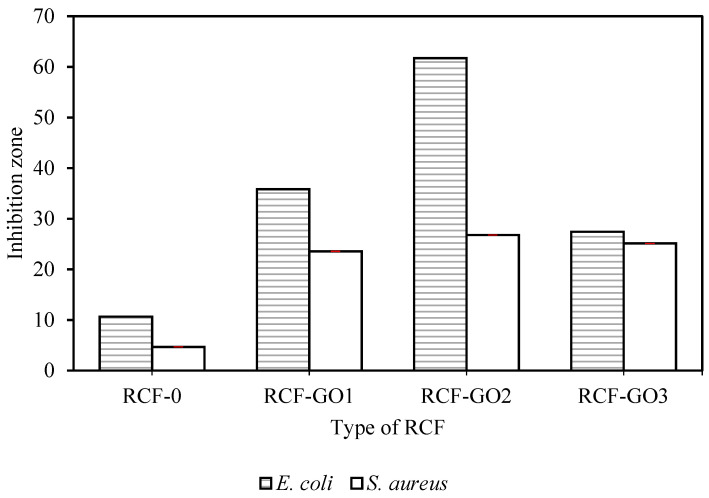
Bacterial inhibition zone of RCFs with and without GO.

**Figure 11:**
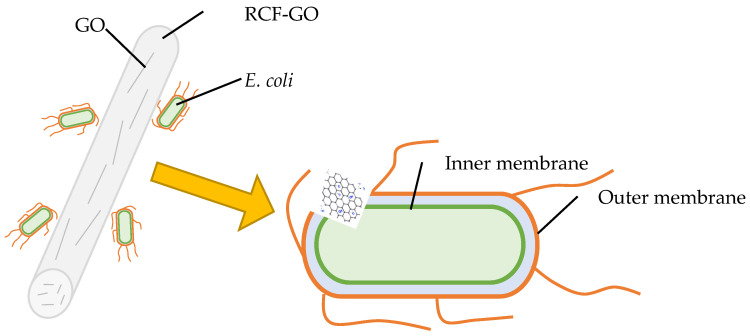
Schematic of the “nano-blade” effect of GO towards a Gram-negative bacterial cell (*E. coli*).

As shown in Figure 10, the smallest inhibition zone was observed for RCF−0. The inhibition zone of RCFs increases with the GO content up to 2 wt.%, and then reduces at 3 wt.% of GO loading. This trend can be observed for *E. coli* and *S. aureus*. The highest inhibition against bacteria was shown by RCF−GO2 for both *E. coli* and *S. aureus* with an inhibition zone of 61.73 and 26.78, respectively. It is theorised that the GO acts as a nano-blade (bacterial inhibitor) when it is directly in contact with the bacteria by damaging the cell membrane and leading to intracellular components leaking and cell death, as shown in a schematic diagram in Figure 11. Nano-blade can be observed in FESEM images for RCF−GO2, where the GO layers are visible in the RCF. Raman spectrum also supports the presence of GO on the surface of RCFs, which contributed to the antibacterial properties of the composite. The nano-blade killing mechanism is one of the possible ways for the RCF to project its antibacterial properties. Nevertheless, several other mechanisms may happen in the systems, including causing oxidative pressure and damage to the bacterial cell membrane via the regeneration of ROS [42,43]. A larger inhibition zone for *E. coli* compared to *S. aureus* can be related to the thicker peptidoglycan layer in Gram-positive bacteria (*S. aureus*), which protects the bacterial cell from full contact with the RCF [3]. However, the reduction in bacterial inhibition for RCF−GO3 may be due to GO agglomeration and imperfection of the GO layering in the RCF, as discussed in Section 3.3. Moreover, the lower loading of the GO resulted in a higher tensile strength of the RCFs, as discussed previously (Section 3.6). However, a high-strength fibre is less effective in expressing its antibacterial properties because most of the GO is embedded in the cellulose matrix. This phenomenon limits the interaction between GO and bacteria and affects its properties. As a result, it reduces the effectiveness of the GO to inhibit bacterial growth. RCF–GO with antibacterial properties may contribute to the stability of the fibre as a wound-dressing material. RCF without antibacterial properties may cause infection and other problems, hence, disturbing the wound-healing process. It has been proven that cellulose with antibacterial properties may increase the healing rate and reduce the infection risk [44].

## 4. Conclusions

The macro-sized RCF–GOs with antibacterial properties were successfully prepared by mixing cellulose and GO at different GO concentrations in a NaOH/urea alkaline system. Intercalation and the formation of hydrogen bonds allowed for the observation of a good dispersion of the GO in the cellulose solution after 30 min of incubation, which is evidence that GO is compatible with cellulose. Raman spectroscopy results showed that GO can only be detected at a higher GO loading. This provides evidence that GO can be found within, as well as on the surface, of the RCF. At high GO concentrations (>2 wt.%), agglomeration led to reduced mechanical, thermal, and antibacterial properties due to the destruction of the intercalation between cellulose and GO. As a potential material for use in biomedical applications, RCF−GO2 (with 2 wt.% GO) demonstrated the best performance in mechanical and thermal properties overall. It also exhibited the best antibacterial properties against both Gram-positive and Gram-negative bacteria.

## Figures and Tables

**Table 1:** The crystallinity index of RCFs with and without GO.

Sample	Crystallinity Index (%)
Cotton linter	58.89
RCF−0	56.94
RCF−GO1	57.44
RCF−GO2	57.74
RCF−GO3	57.58

**Table 2:** Thermal degradation of RCF–GOs.

Sample	T_o_ (°C)	T_max_ (°C)	T_f_ (°C)	Residue (%)
RCF-0	275	322	345	17.98
RCF−GO1	287	328	345	27.22
RCF−GO2	305	328	345	22.21
RCF−GO3	281	322	345	28.24

## Data Availability

Not applicable.
